# Maternal pre-pregnancy overweight and gestational diabetes and dietary intakes among young adult offspring

**DOI:** 10.1038/s41387-020-00129-w

**Published:** 2020-07-23

**Authors:** Nina Kaseva, Marja Vääräsmäki, Hanna-Maria Matinolli, Marika Sipola, Marjaana Tikanmäki, Noora Kanerva, Kati Heinonen, Aulikki Lano, Dieter Wolke, Sture Andersson, Marjo-Riitta Järvelin, Katri Räikkönen, Johan G. Eriksson, Satu Männistö, Eero Kajantie

**Affiliations:** 1 National Institute for Health and Welfare, Helsinki, Oulu, Finland; 2 PEDEGO Research Unit (Research Unit for Pediatrics, Dermatology, Clinical Genetics, Obstetrics and Gynecology), Medical Research Center Oulu (MRC Oulu), Oulu University Hospital and University of Oulu, Oulu, Finland; 3 Research Center for Child Psychiatry, University of Turku, Turku, Finland; 4 INVEST Research Flagship, University of Turku, Turku, Finland; 5 Department of Public Health, University of Helsinki, Helsinki, Finland; 6 Department of Psychology and Logopedics, University of Helsinki, Helsinki, Finland; 7 Children’s Hospital, Pediatric Research Center, University of Helsinki and Helsinki University Hospital, Helsinki, Finland; 8 Department of Psychology, University of Warwick, Warwick, UK; 9 Department of Epidemiology and Biostatistics, MRC–PHE Centre for Environment & Health, School of Public Health, Imperial College London, London, UK; 10 Center for Life Course Health Research, Faculty of Medicine, University of Oulu, Oulu, Finland; 11 Biocenter Oulu, University of Oulu, Oulu, Finland; 12 Unit of Primary Care, Oulu University Hospital, Oulu, Finland; 13 Department of General Practice and Primary Health Care, University of Helsinki and Helsinki University Hospital, Helsinki, Finland; 14 Folkhälsan Research Center, Helsinki, Finland; 15 Department of Obstetrics and Gynecology, National University Singapore, Yong Loo Lin School of Medicine, Singapore, Singapore; 16 Singapore Institute for Clinical Sciences, Agency for Science, Technology and Research (A*STAR), Singapore, Singapore; 17 Department of Clinical and Molecular Medicine, Norwegian University of Science and Technology, Trondheim, Norway

**Keywords:** Malnutrition, Obesity, Risk factors, Gestational diabetes

## Abstract

**Background/Objectives:**

Maternal pre-pregnancy overweight/obesity and gestational diabetes (GDM) are associated with increased fat deposition in adult offspring. The purpose of this study was to identify if maternal pre-pregnancy overweight (body mass index (BMI) ≥ 25 kg/m^2^) or GDM are associated with dietary quality or intake in adult offspring.

**Subjects/Methods:**

Participants (*n* = 882) from two longitudinal cohort studies (ESTER Maternal Pregnancy Disorders Study and the Arvo Ylppö Longitudinal Study) completed a validated food-frequency questionnaire at a mean age of 24.2 years (SD 1.3). Diet quality was evaluated by a Recommended Finnish Diet Index (RDI). The study sample included offspring of normoglycaemic mothers with pre-pregnancy overweight/obesity (ONO = 155), offspring of mothers with GDM regardless of BMI (OGDM = 190) and offspring of mothers with normal weight and no GDM (controls; *n* = 537).

**Results:**

Among men, daily energy and macronutrient intakes were similar in ONO and controls. However, after adjusting for current offspring characteristics, including BMI, daily carbohydrate intake relative to total energy intake was higher in ONO-men [2.2 percentages of total energy intake (95% confidence interval 0.4, 4.0)]. In ONO-women, macronutrient intakes relative to total energy intake were similar with controls, while total daily energy intake seemed lower [−587.2 kJ/day (−1192.0, 4.4)]. After adjusting for confounders, this difference was attenuated. Adherence to a healthy diet, as measured by RDI, was similar in ONO and controls [mean difference: men 0.40 (−0.38, 1.18); women 0.25 (−0.50, 1.00)]. In OGDM vs. controls, total energy and macronutrient intakes were similar for both men and women. Also adherence to a healthy diet was similar [RDI: men 0.09 (−0.62, 0.80); women −0.17 (−0.93, 0.59)].

**Conclusions:**

Our study suggested higher daily carbohydrate intake in male offspring exposed to maternal pre-pregnancy overweight/obesity, compared with controls. Prenatal exposure to GDM was not associated with adult offspring dietary intakes.

## Introduction

Obesity, officially recognized as a disease by the World Health Organization (WHO)^1^, is preventable. Yet, overweight (body mass index, BMI ≥ 25 kg/m^2^) and obesity (BMI ≥ 30 kg/m^2^) affect two billion people globally^2^. In 2016 39% of all adults worldwide were overweight and 13% were obese^2^. The cumulative effect of obesity, extending from childhood and adolescence to midlife, increases the likelihood of complications and death related to diabetes^3^, cardiovascular disease^4^ and cancer^5^.

Obesity is a multifactorial condition, influenced by genes, lifestyle and environmental factors. Approximately 40–70% of variation in obesity is attributed to genetic factors^6–10^. Of lifestyle factors, high physical activity, fitness and low sedentary behavior have all been shown to modify the extent of how known obesity gene variants affect BMI^11^. Further, excess energy intake, socioeconomic factors and educational status, feminine gender and environmental factors are also known to be associated with overweight and obesity^12^.

In addition to genes and current environment, overweight and obesity are affected by prenatal environment. For example, prenatal exposure to a hyperglycaemic environment often caused by maternal overweight/obesity during pregnancy or gestational diabetes (GDM) changes growth trajectories and homeostatic regulatory mechanisms, potentially predisposing the fetus to epigenetic changes^13^. Such changes may cause an increased risk of next generation overweight/obesity through fetal programming. Previous studies have linked high maternal pre-pregnancy BMI to unfavorable offspring body composition in infancy^14^, childhood^15^, adolescence^16^, young^17^ and late adulthood^18^. Also offspring to mothers with GDM show unfavorable body composition by adolescence^19^ and extending into adulthood^17^. Then again, maternal pre-pregnancy healthy lifestyle characterized by normal weight, physical activity, a healthy diet and avoiding smoking is associated with 75% lower risk of offspring obesity in childhood through early adulthood^20^. Some of these associations may be explained by genetic or lifestyle factors shared by family members, while, in part, these findings may represent causal programming effects.

Moreover, previous studies suggest that an unhealthy diet especially in people with obesity or diabetes may affect their epigenome, and consequently disease pathogenesis^21–23^. In addition, the impact of an unhealthy diet on non-communicable disease morbidity and mortality is extensive; 11 million deaths (10 million cardiovascular, 0.9 million cancer and 0.3 million type 2 diabetes) and 255 million disability-adjusted life-years globally in 2017 were attributable to dietary risk factors^24^. One possibility is that the association between prenatal environment and offspring overweight and obesity is mediated through prenatal programming of food intake.

We assessed habitual diet by a validated food-frequency questionnaire (FFQ) in young adult offspring born to mothers with pre-pregnancy overweight/obesity or GDM and controls, not exposed to these maternal pregnancy conditions. We hypothesized that offspring exposure to maternal pre-pregnancy overweight/obesity or GDM predicts unhealthy offspring dietary intake as observed in adult age.

## Materials/subjects and methods

### Study population

**Fig. 1:**
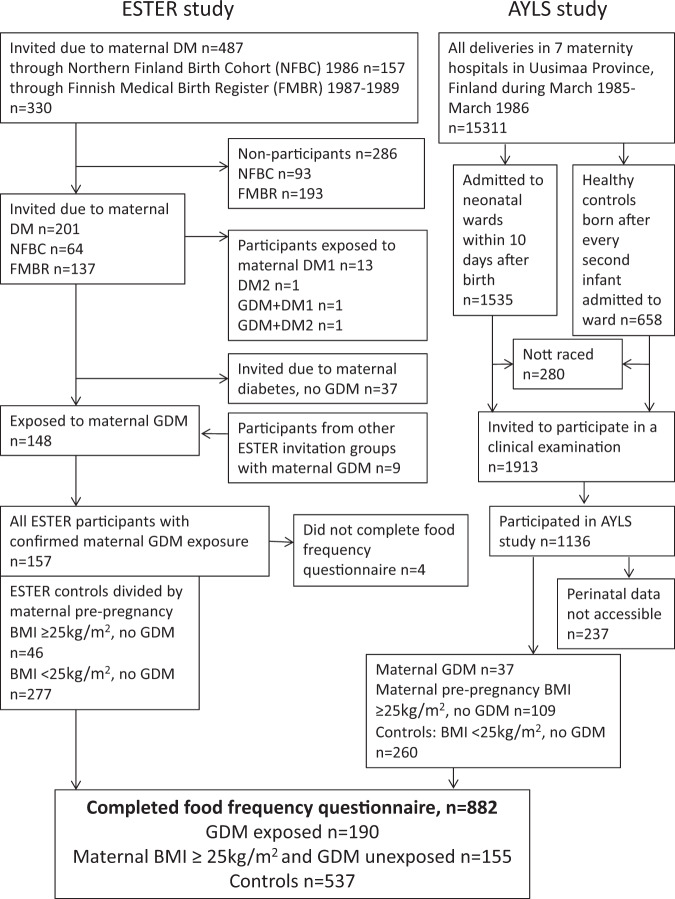
Study population. Flow chart of the study population, including participants from two birth cohort studies.

The study participants come from two prospective birth cohorts (Fig. 1): the ESTER Maternal Pregnancy Disorders Study^25,26^ and the Arvo Ylppö Longitudinal Study (AYLS)^27,28^. The ESTER Study consists of two arms (Fig. 1): (1) ESTER Preterm Birth^25^ and (2) ESTER Maternal Pregnancy Disorders arms. The present study includes participants from the latter arm. All ESTER study participants were born in the two northernmost provinces of Finland. Those born in 1985–1986 were recruited from the Northern Finland Birth Cohort 1986 (ref. ^26^) and those born in 1987–1989 through the Finnish Medical Birth Register^25^. We selected all participants who were confirmed to have maternal GDM (*n* = 157), regardless of the mother’s pre-pregnancy BMI. Among ESTER participants originally invited as controls, participants were stratified into two groups: (1) offspring born at term to mothers with pre-pregnancy BMI ≥ 25 kg/m^2^ and no GDM (*n* = 46) and (2) the control group constituted the remaining controls, all with maternal pre-pregnancy BMI < 25 kg/m^2^ and no GDM (*n* = 277).

All AYLS participants were born in the province of Uusimaa, in Southern Finland between 1985 and 1986 (Fig. 1). This cohort consists of all live-born infants admitted to neonatal wards in obstetric units, or transferred to the neonatal intensive care unit of the Children’s Hospital, Helsinki University Central Hospital within 10 days of their birth. This cohort population ranges from severely ill preterm infants to infants born at term, requiring only brief inpatient observation, and their controls^27,28^. From the AYLS cohort we selected all participants who were (1) exposed to maternal GDM at any maternal BMI (*n* = 37), (2) those who had maternal BMI ≥ 25 kg/m^2^ and no GDM (*n* = 109) and (3) controls (i.e. originally recruited as controls, maternal BMI < 25 kg/m^2^ and no GDM; *n* = 260).

Perinatal data were collected from healthcare records and questionnaires. Length of gestation, maternal GDM, hypertension (gestational or chronic) and pre-eclampsia (including superimposed) diagnoses were independently confirmed according to prevailing criteria by reviewing original hospital records^19,29^. Maternal GDM was screened for and diagnosed by a 2-h oral glucose tolerance test (OGTT) in maternal welfare clinics between 26 and 28 gestational weeks. Indications for screening were glycosuria, prior GDM, suspected fetal macrosomia, previous macrosomic infant (birth weight >4500 g), maternal pre-pregnancy BMI ≥ 25 kg/m^2^ and maternal age ≥40 years. The OGTT was performed after overnight fasting by using a 75-g oral glucose load. At the time of diagnosis in the 1980s, the following cut-off points for GDM were used for venous blood glucose: >5.5 mmol/l at fasting, >11.0 and >8.0 mmol/l, 1 and 2 h after the glucose load, respectively. According to prevailing national guidelines, a diagnosis of GDM required a minimum of one abnormal value in the OGTT^19^. For comparison, the International Association of Diabetes and Pregnancy Study Groups (IADPSG) Consensus Panel diagnostic criteria used today are set at fasting plasma glucose ≥5.1 mmol/l, and ≥10.0 and ≥8.5 mmol/l following a 75-g oral glucose load^30^.

Offspring to mothers with type 1 (*n* = 28) or 2 diabetes (*n* = 1) were excluded from all analyses. We further excluded participants who were pregnant (*n* = 9) during the clinical examination, reported having cerebral palsy (*n* = 8), mental disability (*n* = 11) or severe physical disability (*n* = 5), as these conditions might affect the measured outcomes. All ESTER and AYLS cohort participants who completed the FFQ were categorized into three groups: (1) offspring of mothers with GDM (OGDM) at any level of maternal BMI, (2) offspring of normoglycaemic mothers with pre-pregnancy overweight/obesity (ONO) and (3) controls, i.e. offspring of mothers with pre-pregnancy BMI < 25 kg/m^2^ and no GDM. As a result, 882 subjects were included in the analyses; OGDM *n* = 190, ONO *n* = 155 and controls *n* = 537 (Fig. 1).

### Ethics

This study protocol was performed in accordance with the Declaration of Helsinki. The ethics committees of the University of Oulu, Helsinki City Maternity Hospital, Helsinki University Central Hospital, Jorvi Hospital and Northern Ostrobothnia Hospital District, and the Coordinating Ethics Committee of the Helsinki and Uusimaa Hospital District approved this study. All study participants provided written informed consent. Because of individual participant consent, these data are not freely available. Investigators requesting data access are asked to contact the corresponding author. Requests may be subject to ethics review or participant consent, or both.

### Data collection

Clinical examinations including questionnaires and measures were conducted in 2009–2011 for ESTER participants and in 2009–2012 for AYLS participants. Questionnaires about smoking, current medications, medical history and health status were completed by the participants. Highest parental education was recorded and categorized into four levels (dummy coded as basic, secondary, lower- and upper-level tertiary) to serve as an indicator of childhood socioeconomic status. Participants were measured without socks and shoes, wearing light clothes. Height was measured three times with a portable stadiometer. Weight was measured with an electronic scale. BMI was calculated as weight (kg) divided by height (m) squared. Waist circumference (midway between the lowest rib and the iliac crest) was measured twice with means of the results used in the analyses. Height and waist circumference were measured to the nearest 0.1 cm and weight to the nearest 0.1 kg.

### Assessment of dietary intake and diet quality

Habitual diet was assessed using a validated, semiquantitative FFQ^31^, including 131 commonly consumed food and beverage items. The FFQ was designed to cover diet over the preceding 12 months. Participants were asked to complete the FFQ at the study site, and it was subsequently reviewed by a trained study nurse. Participants indicated frequency of consuming each food item with nine categories ranging from “never or seldom” to “six or more times daily”. Portion size was fixed separately for men and women, using natural units if applicable (e.g. slice, glass).

We calculated average daily energy, macronutrient and food intakes using a Finnish food composition database, Fineli®^32^. Total energy intake is expressed in kJ/day, while macronutrient intakes are shown as percentages of total energy intake (*E*%).

For assessment of diet quality we calculated the Recommended Finnish Diet Index (RDI)^33^. The RDI is based on Finnish nutrition recommendations^34^ and it summarizes information on the following dietary factors: average daily consumption of fruits and berries; vegetables; rye; salt; sucrose; alcohol; ratio of white meat to red and processed meat; ratio of polyunsaturated fatty acids to saturated fatty acids and trans-fatty acids. The RDI score was calculated according to quartiles of consumption of each dietary component by adding the sum of points given (0 or 1 for alcohol and 0–3 for the remaining components). Regarding the ratio of white meat to red and processed meat vegetarians were given 3 points. A maximum score of 22 includes all measured RDI components, and when excluding alcohol consumption the maximum RDI score is 21, with higher score indicating healthier diet.

### Statistical analysis

Statistical analyses were performed using IBM SPSS Statistics versions 25 and 26 (IBM Corp., Armonk, NY). Analyses were conducted using a combined data set of the two birth cohorts (ESTER and AYLS). We compared population characteristics between participants by mean values, using *T*-tests (continuous variables) and *χ*^2^-tests (categorical variables). Variation within groups is described by SDs and the power of sample size is indicated by confidence intervals.

Before performing any statistical analyses, we log-transformed [ln (*x* + 1)] intakes of rye, fruits and vegetables, as these data were not normally distributed. Therefore, all mean differences in these food intakes are reported as back-transformed percentages.

We used linear regression models to compare macronutrient and diet intake between adult offspring of mothers with pre-pregnancy overweight/obesity or GDM with controls. In analyses comparing total energy and macronutrient intakes, we adjusted for age and cohort in model 1. In model 2 we included prenatal and parental confounders: age, cohort, parental education, birth weight SD score, gestational age, maternal smoking during pregnancy, maternal hypertension during pregnancy and pre-eclampsia. In model 3 we further considered participant-related factors: BMI, smoking and living at parental home were added to the covariates of model 2. In all analyses comparing RDI and its components, total energy intake was additionally included in models 1–3. Results are shown as mean differences (95% CIs) and presented separately for men and women.

### Sensitivity analyses to account for underreporting

The FFQ is a validated method for estimating dietary intake^31,35,36^. However, people tend to misreport or provide inaccurate estimates of their food consumption^31,37,38^. Sex, age, educational level, smoking, area of living, weight status and desire for weight change are all factors known to correlate with underreporting^31,37,38^. Based on the Goldberg cut-off value (≤1.14), for the ratio of reported energy intake to basal metabolic rate^39^, we therefore estimated the proportion of underreporting. To explore whether our results were affected by underreporting, we reran all analyses comparing ONO and OGDM groups with controls after excluding the underreporting participants. Further, including all participants, we additionally adjusted for underreporting in models 1–3 and then reran all analyses.

## Results

The study sample included 882 participants (51% women) (Fig. 1) whose perinatal and current characteristics are presented in Table 1. Mothers with GDM or pre-pregnancy overweight/obesity had higher BMIs at start of pregnancy, they more often presented with hypertension and their offspring was born large for gestational age more often, while their duration of pregnancy was shorter than in controls. In addition, OGDM had higher birth weights and more siblings, while ONO were more often born preterm or small for gestational age, compared with controls. ONO and OGDM participants had higher BMIs and waist circumferences than the controls and OGDM participants lived more often with their parents. Parental educational attainment and weight gain during pregnancy were highest in the control group. We reran all analyses after excluding twins (*n* 12); this did not change our results (data not shown). We also performed subanalyses, additionally adjusting for maternal gestational weight gain in models 2 and 3. This did not change our results (data not shown).

**Table 1:** Participant characteristics of adult offspring exposed to maternal gestational diabetes (regardless of maternal body mass index), maternal pre-pregnancy obesity or overweight without gestational diabetes, and their controls, i.e. offspring of normoglycaemic mothers with normal pre-pregnancy weight.

Characteristics	Maternal gestational diabetes (*n* = 190)	Maternal pre-pregnancy obesity or overweight^a^, no gestational diabetes (*n* = 155)	Controls (*n* = 537)
*Birth/perinatal characteristics*			
Maternal body mass index before pregnancy, mean (SD), kg/m^2^	25.0 (5.5)^†^	28.0 (3.0)^†^	21.2 (1.9)
Maternal weight gain during pregnancy, mean (SD), kg	11.8 (5.4)^†^	13.3 (5.5)*	14.1 (4.3)
Maternal body mass index ≥25 kg/m^2^ before pregnancy, *n* (%)	72 (37.9)	155 (100.0)	NA
Maternal body mass index ≥30 kg/m^2^ before pregnancy, *n* (%)	29 (15.3)	27 (17.4)	NA
Maternal multiparity, *n* (%)	130 (68.4)^†^	86 (55.5)	329 (61.3)
Twin pregnancy, *n* (%)	2 (1.1)	2 (1.3)	8 (1.5)
Maternal hypertension, *n* (%)	39 (20.5)^†^	45 (29.0)^†^	48 (8.9)
Maternal pre-eclampsia, *n* (%)	12 (6.3)	8 (5.2)	17 (3.2)
Maternal smoking during pregnancy, *n* (%)	22 (11.6)	32 (20.6)	87 (16.2)
Birth weight, mean (SD), g	3696 (603)^**^	3463 (876)	3538 (465)
Birth weight SD score, mean (SD)	0.55 (1.2)^†^	0.10 (1.4)	−0.04 (0.9)
Gestational age, mean (SD), weeks	39.0 (1.6)^†^	39.1 (2.9)*	39.9 (1.4)
Born preterm, *n* (%)^b^	18 (9.5)	27 (17.4)**	18 (3.4)
Small for gestational age, *n* (%)	1 (0.5)	15 (9.7)^†^	8 (1.5)
Large for gestational age, *n* (%)	25 (13.2)^†^	11 (7.1)*	10 (1.9)
Men, *n* (%)	104 (54.7)	75 (48.4)	252 (46.9)
*Current characteristics*			
Age, mean (SD), years	23.4 (1.4)^†^	24.8 (1.1)^†^	24.4 (1.3)
Daily smoking, n (%)	55 (28.9)	52 (33.5)	139 (25.8)
Body mass index, mean (SD), kg/m^2^			
Men	25.7 (4.7)**	26.5 (4.9)^†^	24.0 (3.6)
Women	24.3 (4.4)*	24.7 (4.6)**	23.1 (4.3)
Body mass index ≥25 kg/m^2^, *n* (%)	80 (42.1)**	75 (48.4)^†^	155 (28.9)
Body mass index ≥30 kg/m^2^, *n* (%)	25 (13.2)*	22 (14.2)*	39 (7.3)
Height, mean (SD), cm			
Men	178.8 (6.7)	180.2 (7.0)	178.9 (7.0)
Women	165.9 (6.3)	164.8 (6.4)	165.2 (6.3)
Waist circumference, mean (SD), cm			
Men	88.1 (12.2)**	90.7 (12.4)^†^	84.1 (9.0)
Women	79.2 (10.8)**	80.1 (11.1)**	75.7 (9.8)
Currently living at parental home, *n* (%)	32 (16.8)**	11 (7.1)	43 (8.0)
Parental education, *n* (%)	^†^	**	
Basic	29 (15.3)	21 (13.5)	29 (5.4)
Secondary	100 (52.6)	80 (51.6)	266 (49.5)
Lower-level tertiary	15 (7.9)	19 (12.3)	64 (11.9)
Upper-level tertiary	44 (23.2)	28 (18.1)	165 (30.7)
*Macronutrient intake*			
Total energy intake, mean (SD), kJ/day			
Men	11056 (5256)	10290 (3732)	10703 (3624)
Women	7716 (2375)	7381 (2084)	7952 (2403)
Carbohydrate, mean (SD), *E*%			
Men	43.9 (6.6)	45.7 (6.5)	44.4 (6.1)
Women	46.2 (6.9)	47.3 (7.5)	47.2 (6.9)
Sucrose, mean (SD), *E*%			
Men	8.6 (3.3)	9.5 (3.7)	8.9 (3.5)
Women	10.4 (3.7)	10.4 (3.4)	11.0 (4.4)
Fat, mean (SD), *E*%			
Men	34.3 (4.8)	32.7 (4.3)	33.9 (4.9)
Women	33.5 (5.3)	32.8 (6.2)	33.0 (5.6)
Protein, mean (SD), *E*%			
Men	19.1 (3.3)	18.5 (3.5)	18.6 (3.1)
Women	18.6 (2.5)*	17.7 (2.5)	18.0 (2.6)
Alcohol, mean (SD), *E*%			
Men	2.7 (3.5)	3.1 (3.1)	3.1 (3.2)
Women	1.7 (2.6)	2.1 (2.6)	1.9 (2.5)
Recommended Diet Index			
Men	11.5 (2.6)	12.0 (2.6)	11.5 (3.0)
Women	11.4 (2.8)	11.7 (2.8)	11.4 (3.0)

*E%* percent of total energy intake.

*p value <0.05; **p value <0.01.

^†^*p* value <0.001 (*T*-test for continuous and *χ*^2^ test for categorical variables, comparing offspring exposed to maternal gestational diabetes or maternal pre-pregnancy overweight/obesity with controls. All remaining *p* values are >0.05).

^a^Pre-pregnancy body mass index ≥25 kg/m^2^.

^b^Gestational age <37 weeks.

### Dietary intake in offspring to normoglycaemic mothers with pre-pregnancy overweight (ONO-participants)

Macronutrient data are presented in Tables 1 (unadjusted) and 2 (adjusted). Among ONO-men, daily energy and macronutrient intakes were similar to controls (Tables 1 and 2). However, after adjusting for confounders and current offspring characteristics (model 3, including age, cohort, parental education, perinatal factors, participant BMI, smoking, and living at parental home), daily carbohydrate intake was 2.2*E*% (95% CI: 0.4, 4.0) higher in ONO-men compared with controls (Table 2).

**Table 2:** Macronutrient intake of young adults exposed to maternal gestational diabetes (regardless of maternal body mass index) or maternal pre-pregnancy overweight or obesity without gestational diabetes, compared with controls, i.e. offspring to normoglycaemic mothers with normal pre-pregnancy weight.

	Maternal gestational diabetes (*n* = 190)				Maternal pre-pregnancy overweight or obesity, no gestational diabetes (*n* = 155)			
	Men		Women		Men		Women	
	Mean difference	95% CI	Mean difference	95% CI	Mean difference	95% CI	Mean difference	95% CI
*Energy (kJ/day)*								
Model 1	309	−697, 1315	−183	−804, 438	−511	−1475, 454	−598	−1192, −4^*^
Model 2	341	−779, 1460	−174	−848, 500	−637	−1696, 421	−656	−1319, 8
Model 3	213	−924, 1350	−68	753, 618	−819	−1907, 269	−583	−1254, 88
*Carbohydrate (E%)*								
Model 1	−0.7	−2.2, 0.8	−1.3	−3.1, 0.5	1.5	−0.1, 3.1	0.4	−1.4, 2.2
Model 2	−0.6	−2.3, 1.1	−1.4	−3.3, 0.6	1.1	−0.7, 2.9	0.7	−1.3, 2.7
Model 3	−0.4	−2.0, 1.3	−1.1	−3.1, 0.8	2.2	0.4, 4.0^*^	0.8	−1.1, 2.8
*Sucrose (E%)*								
Model 1	−0.2	−1.0, 0.6	−0.2	−1.3, 0.9	0.6	−0.3, 1.5	−0.7	−1.8, 0.3
Model 2	−0.1	−1.0, 0.8	0.0	−1.1, 1.2	0.5	−0.5, 1.6	−0.3	−1.5, 0.8
Model 3	0.1	−0.8, 1.0	0.1	−1.1, 1.3	0.9	−0.1, 2.0	−0.3	−1.5, 0.9
*Fat (E%)*								
Model 1	0.2	−0.9, 1.4	0.5	−0.9, 2.0	−1.0	−2.2, 0.3	−0.3	−1.7, 1.2
Model 2	0.4	−1.0, 1.7	1.0	−0.5, 2.6	−0.6	−2.0, 0.7	−0.4	−2.1, 1.2
Model 3	0.3	−1.0, 1.6	0.9	−0.7, 2.5	−1.1	−2.5, 0.3	−0.4	−2.0, 1.3
*Protein (E%)*								
Model 1	0.1	−0.6, 0.7	0.6	−0.1, 1.2	−0.1	−1.0, 0.7	−0.2	−0.8, 0.5
Model 2	−0.1	−0.9, 0.7	0.6	−0.1, 1.3	−0.2	−1.1, 0.7	−0.2	−0.9, 0.5
Model 3	−0.4	−1.2, 0.5	0.6	−0.1, 1.3	−0.6	−1.5, 0.3	−0.3	−1.0, 0.4
*Alcohol (E%)*								
Model 1	0.3	−0.4, 1.1	0.2	−0.5, 0.8	−0.4	−1.2, 0.4	0.1	−0.6, 0.7
Model 2	0.3	−0.5, 1.2	−0.3	−0.9, 0.4	−0.2	−1.2, 0.7	−0.1	−0.8, 0.6
Model 3	0.4	−0.4, 1.3	−0.4	−1.0, 0.3	−0.5	−1.5, 0.4	−0.1	−0.8, 0.6

*E%* percent of total energy intake.

Linear regression models adjusted as follows:

Model 1: adjusted for age and cohort.

Model 2: adjusted in addition for parental education, birth weight SD score, gestational age, maternal smoking during pregnancy, maternal hypertension during pregnancy and pre-eclampsia.

Model 3: adjusted in addition for offspring BMI, smoking and living at parental home.

*p value <0.05.

In ONO-women, daily energy intake was lower in model 1, although adjusting for confounders and current offspring characteristics attenuated this difference (Table 2). All macronutrient intakes were similar between ONO-women and controls.

RDI and consumption of its components are shown in Tables 3 and 4. Both adherence to the recommended diet and intakes of its components were similar for ONO and control participants.

**Table 3:** Recommended diet intake index of young adults exposed to maternal gestational diabetes (regardless of maternal body mass index) or maternal pre-pregnancy overweight or obesity without gestational diabetes, compared with controls, i.e. offspring to normoglycaemic mothers with normal pre-pregnancy weight.

	Maternal gestational diabetes (*n* = 190)				Maternal pre-pregnancy overweight or obesity, no gestational diabetes (*n* = 155)			
	Men		Women		Men		Women	
	Mean difference	95% CI	Mean difference	95% CI	Mean difference	95% CI	Mean difference	95% CI
*Recommended Diet Index*^a^								
Model 1	0.09	−0.62, 0.80	−0.17	−0.93, 0.59	0.40	−0.38, 1.18	0.25	−0.50, 1.00
Model 2	−0.14	−0.91, 0.64	−0.12	−0.94, 0.70	0.29	−0.57, 1.14	0.23	−0.60, 1.06
Model 3	−0.03	−0.81, 0.75	−0.14	−0.96, 0.68	0.47	−0.41, 1.35	0.08	−0.76, 0.92

Linear regression models adjusted as follows:

Model 1: adjusted for age, cohort and total energy intake.

Model 2: adjusted in addition for parental education, birth weight SD score, gestational age, maternal smoking during pregnancy, maternal hypertension during pregnancy and pre-eclampsia.

Model 3: adjusted in addition for offspring BMI and smoking, and living at parental home.

^a^Maximum score 21 (alcohol intake not included), with higher score indicating a healthier diet.

**Table 4:** Mean differences (95% CIs) of the components of the recommended diet index, comparing young adults exposed to maternal gestational diabetes (regardless of maternal body mass index) or maternal pre-pregnancy overweight or obesity without gestational diabetes, compared with controls, i.e. offspring to normoglycaemic mothers with normal pre-pregnancy weight.

	Men					Women				
	Controls	Maternal gestational diabetes		Maternal pre-pregnancy overweight or obesity, no gestational diabetes		Controls	Maternal gestational diabetes		Maternal pre-pregnancy overweight or obesity, no gestational diabetes	
	Mean (SD)	Mean difference (95% CI)	*p*	Mean difference (95% CI)	*p*	Mean (SD)	Mean difference (95% CI)	*p*	Mean difference (95% CI)	*p*
*Fat ratio*^a^	0.46 (0.13)					0.46 (0.14)				
Model 1		−0.00 (−0.04, 0.03)	0.80	0.00 (−0.03, 0.04)	0.93		−0.02 (−0.06, 0.01)	0.18	0.00 (−0.03, 0.04)	0.91
Model 2		−0.01 (−0.04, 0.03)	0.78	0.00 (−0.04, 0.04)	0.91		−0.02 (−0.05, 0.02)	0.39	0.01 (−0.03, 0.04)	0.77
Model 3		0.00 (−0.03, 0.04)	0.97	0.01 (−0.03, 0.04)	0.81		−0.01 (−0.05, 0.02)	0.49	0.00 (−0.04, 0.04)	0.93
*Salt (mg)*	9690 (3570)					6786 (2211)				
Model 1		−129 (−502, 244)	0.50	−25 (−435, 386)	0.91		167 (−109, 444)	0.24	24 (−237, 285)	0.86
Model 2		−122 (−538, 294)	0.56	−141 (−596, 313)	0.54		156 (−142, 454)	0.30	3.1 (−287, 293)	0.98
Model 3		−203 (−617, 211)	0.34	−306 (−764, 151)	0.19		132 (−165, 430)	0.38	−8.5 (−297, 280)	0.95
*Sucrose (E%)*	8.9 (3.5)					11.0 (4.4)				
Model 1		−0.21 (−1.03, 0.62)	0.62	0.65 (−0.28, 1.58)	0.17		−0.20 (−1.30, 0.90)	0.72	−0.62 (−1.68, 0.46)	0.26
Model 2		−0.14 (−1.07, 0.78)	0.76	0.56 (−0.47, 1.59)	0.28		0.05 (−1.13, 1.23)	0.93	−0.19 (−1.38, 0.99)	0.75
Model 3		0.06 (−0.84, 0.96)	0.90	1.00 (−0.03, 2.03)	0.06		0.07 (−1.12, 1.26)	0.91	−0.13 (−1.33, 1.06)	0.83
*Meat ratio*^b^	0.68 (0.6)					1.2 (1.9)				
Model 1		−0.04 (-0.16, 0.08)	0.50	0.17 (−0.09, 0.43)	0.20		0.29 (−0.25, 0.83)	0.29	0.38 (−0.26, 1.01)	0.25
Model 2		−0.06 (−0.19, 0.08)	0.39	0.12 (−0.16, 0.40)	0.39		0.53 (−0.07, 1.12)	0.08	0.55 (−0.17, 1.27)	0.13
Model 3		−0.06 (−0.20, 0.08)	0.39	0.13 (−0.17, 0.42)	0.41		0.59 (−0.01, 1.19)	0.06	0.58 (−0.15, 1.31)	0.12
*Rye (%)*^c^	35.7 (31.4)					37.0 (29.1)				
Model 1		−7.92 (−27.12, 16.33)	0.49	−19.11 (−7.32, 53.06)	0.17		−2.26 (−23.89, 25.51)	0.86	9.90 (−14.70, 41.60)	0.46
Model 2		−12.40 (−32.40, 13.51)	0.32	6.25 (−19.30, 39.89)	0.67		−3.53 (−26.49, 26.60)	0.80	2.13 (−22.99, 35.43)	0.88
Model 3		−13.81 (−33.93, 12.44)	0.27	8.25 (−18.89, 44.48)	0.59		−1.85 (−25.38, 29.09)	0.89	−1.00 (−25.38, 31.35)	0.94
*Fruits (%)*^c^	155.0 (156.3)					214.2 (177.6)				
Model 1		−4.11 (−24.32, 21.51)	0.28	11.48 (−12.68, 42.32)	0.38		1.09 (−18.50, 25.38)	0.92	5.94 (−14.99, 32.03)	0.61
Model 2		−3.76 (−25.99, 25.15)	0.78	11.41 (−14.92, 45.89)	0.43		4.55 (−16.48, 30.87)	0.70	3.77 (−18.16, 31.58)	0.76
Model 3		1.33 (−22.22, 32.02)	0.92	24.86 (−5.01, 64.13)	0.11		6.32 (−14.84, 32.75)	0.59	0.31 (−20.67, 26.82)	0.98
*Vegetables (%)*^c^	226.4 (164.7)					247.6 (158.7)				
Model 1		2.37 (−12.67, 19.99)	0.77	−2.26 (−17.39, 15.64)	0.79		5.80 (−9.40, 23.55)	0.48	−2.59 (−17.10, 14.45)	0.75
Model 2		−6.88 (−21.57, 10.56)	0.42	−4.00 (−19.98, 15.15)	0.66		11.82 (−5.44, 32.24)	0.19	5.06 (−11.97, 25.39)	0.58
Model 3		−2.63 (−18.01, 15.63)	0.76	1.55 (−15.65, 22.27)	0.87		11.08 (−6.39, 31.80)	0.23	3.07 (−13.85, 23.31)	0.74
*Alcohol (E%)*	3.1 (3.2)					1.9 (2.5)				
Model 1		0.32 (−0.44, 1.07)	0.41	−0.40 (−1.22, 0.42)	0.33		0.13 (−0.50, 0.76)	0.68	−0.06 (−0.68, 0.56)	0.85
Model 2		0.34 (−0.50, 1.19)	0.43	−0.29 (−1.20, 0.63)	0.54		−0.31 (−0.94, 0.33)	0.34	−0.23 (−0.93, 0.47)	0.51
Model 3		0.43 (−0.44, 1.29)	0.33	−0.57 (−1.52, 0.38)	0.24		−0.37 (−1.02, 0.28)	0.27	−0.22 (−0.93, 0.49)	0.54

Linear regression models adjusted as follows:

Model 1: adjusted for age, cohort and total energy intake.

Model 2: adjusted in addition for parental education, birth weight SD score, gestational age, maternal smoking during pregnancy, maternal hypertension during pregnancy and pre-eclampsia.

Model 3: adjusted in addition for offspring BMI and smoking, and living at parental home

*E%* percent of total energy intake.

^a^Ratio of polyunsaturated fatty acids to saturated fatty acids and trans-fatty acids.

^b^Ratio of white meat to red and processed meat.

^c^Components expressed as percentages are back-transformed percentages from log-transformed values.

### Dietary intake in offspring to mothers with gestational diabetes (OGDM participants)

In both men and women, macronutrient intakes were similar in OGDM and control participants (Tables 1 and 2). Also adherence to the recommended diet and its components were similar in OGDM and controls (Tables 3 and 4).

### Sensitivity analyses excluding the under-reporters

When stratified by group, 49.7% of ONO, 41.6% of OGDM and 35.4% of controls were underreporting (*n* = 346) based on the Goldberg cut-off value (≤1.14)^39^.

In ONO (both men and women) and OGDM-women, energy intake was not different compared with controls, while OGDM-men had higher energy intake [model 1: 1342 kJ/day (125, 2559, *p* 0.031)] than controls. This difference attenuated after adjusting for confounders in models 2 and 3.

Compared with controls, daily carbohydrate intake was higher in both ONO-men [model 1: 2.1*E*% (0.0, 4.1; *p* 0.047)] and ONO-women [model 1: 3.2*E*% (1.0, 5.4; *p* 0.006)], while fat intake was lower [model 1: men −1.6*E*% (−3.2, 0.1; *p* 0.058), women −3.0*E*% (−4.8, −1.2; *p* 0.001)]. No differences were seen in intakes of sucrose, protein or alcohol between ONO and controls.

Adherence to the recommended diet was similar between groups. However, in comparisons regarding the components of the RDI, ONO consumed more fruits [men: 44.4% (2.5, 103.4; *p* 0.036), women: 42.8% (6.1, 92.1; *p* 0.019)]. In ONO-men, this difference attenuated after further adjustments in models 2 and 3. Furthermore, intake of vegetables was higher in ONO-women compared with controls (model 1: 12.6% (−9.0, 39.1; *p* 0.270), model 2: 28.8% (2.8, 61.3; *p* 0.028), model 3: 27.1 (1.0, 59.9; *p* 0.041)].

Compared with controls, OGDM-participants had similar macronutrient intakes and adherence to the recommended diet. Also comparisons regarding the components of the RDI were similar between OGDM and control groups.

Including all study participants (*n* = 882), we also reran our analyses (models 1–3) with additionally adjusting for underreporting. In ONO-men vs. controls, daily carbohydrate intake was significantly higher [1.6*E*% (95% CI 0.0, 3.3; *p* 0.049)]. This difference increased somewhat after full adjustment in model 3 [2.3*E*% (95% CI 0.5, 4.1; *p* 0.011)]. Otherwise all our results remained similar for ONO and OGDM vs. controls when adjusting for underreporting.

## Discussion

With data combined from two birth cohorts, we assessed food and nutrient intakes in the adult offspring to mothers with overweight/obesity at the start of pregnancy or GDM during the index pregnancy. While our study was adequately powered, we found few differences between the groups. The main difference was that men whose mothers had pre-pregnancy overweight/obesity had as adults higher daily carbohydrate intake than control men. When accounting for underreporting, women whose mothers had pre-pregnancy overweight/obesity had lower fat intake and higher carbohydrate intake, with higher consumption of fruits and vegetables. As for offspring of mothers with GDM, daily energy and macronutrient intakes and dietary habits were similar with controls.

An unhealthy diet is one of many underlying causes of overweight and obesity. In this study we evaluated associations of maternal pre-pregnancy overweight/obesity and GDM on adult offspring diet, a novel approach as data on this subject are scarce. Our finding of higher daily carbohydrate intake in the adult male offspring exposed to maternal pre-pregnancy overweight/obesity is noteworthy, as carbohydrates increase blood glucose levels, stimulating insulin release and accumulating fat tissue especially if consumed in high amounts.

What we see in our findings is a photo at a certain moment. The higher daily intake of carbohydrates in ONO-men vs controls is not necessarily explained by maternal pre-pregnancy BMI. Our finding of higher daily intake of carbohydrates in ONO-men reached statistical significance only when we accounted for current participant-related factors, including participant BMI, smoking and living at parental home. Similarly, among women exposed to maternal pre-pregnancy overweight/obesity, daily carbohydrate intake was higher when underreporting was accounted for. However, when we accounted for underreporting, these women had higher intakes of fruits and vegetables, implying that at least a part of the consumed carbohydrates were other than simple carbohydrates. Fruits and vegetables constitute an important element of a healthy diet. Almost 3% of total mortality in the world is attributable to low intake of fruits and vegetables, and in high-income countries low intake of fruits and vegetables is ranked as the seventh leading risk factor for mortality by WHO^40^.

Accumulating evidence supports an impact of the intrauterine environment in the origins of offspring obesity. Studies have linked prenatal exposure to both high maternal pre-pregnancy BMI and GDM with an unfavorable body composition in the offspring^14–19^. Therefore our finding of lower daily fat intake, especially in ONO-women, is positive, as food fat content and quality, energy density and added sugars are all factors to be emphasized when evaluating food and nutrient intake.

There are several examples showing that pre- or perinatal environment predict later dietary habits^41^. In adults born preterm, lower consumption of vegetables and fruits have been observed^42^. Also among children born at term, an inverse association between fat intake and birth weight has been described^43,44^. Shultis et al. reported inverse associations between birth weight and fat, saturated fat, and protein intake and a positive association between birth weight and carbohydrate intake (all adjusted for energy intake), in early childhood, much of this was explained by confounding factors^44^. These associations were no longer present at 7 years of age^44^. Similarly, in another study with term-born adults, aged 56–70 years, those born with smaller birth size had higher intake of fat and lower intake of carbohydrates^45^. These data suggest that intrauterine growth may modify food intake later in life. In a sense low birth weight or preterm birth, both related to fetal/infant undernutrition, represent opposite exposures than maternal GDM or pre-pregnancy overweight/obesity, which are generally associated with over nutrition. Higher intake of carbohydrates (ONO vs controls) and fruits and vegetable (ONO-women vs controls) could represent the “other end of the spectrum”. Although the excess intake of carbohydrates could be, at least in part, responsible for the greater weight in the ONO group, this does not seem to be the case in the OGDM group. Our finding of higher intakes of carbohydrates in ONO is contrary to observations in other models of early life origins of obesity such as that of children with low birth weight^45^.

### Strengths and limitations of the study

Combining data from two birth cohorts provided a reasonable sample size and we were able to adjust for important confounders, including current participant characteristics and perinatal factors.

All methods assessing habitual diet have weaknesses. We used a validated, self-report FFQ for estimating dietary intake^31,35,36^. The FFQ was filled in by participants at our study site and reviewed by a trained nurse. A known limitation of all self-report methods estimating dietary intake, including the FFQ, is that people tend to underestimate foods considered unhealthy and overestimate those that regarded healthy^31^. High dietary consciousness is known to bias reported food intake in dietary surveys^38^. A larger proportion of underreporting in the ONO group is consistent with this. Underreporting is especially effected by sex, age and BMI^35–38^, all factors we accounted for in our analyses. To further minimize the effects of underreporting on our results, we accounted for underreporting by state of the art methods. This marginally strengthened our findings, although most results remained unchanged. While our study had adequate power to confirm or exclude moderate or large differences, we will not be able to exclude small differences between groups.

Variables that might influence habitual diet include eating disorders, physical activity and sedentary lifestyle. These factors we were unavailable to account for in our study.

### Main findings

Our findings indicate that young adults who were exposed to maternal overweight, obesity or GDM in utero have largely similar nutrient indices and health diet patterns than those not exposed. However, men exposed to maternal overweight/obesity had higher daily carbohydrate intakes in the Finnish population studied.
